# Died of Acute Pneumonia

**Published:** 1891-05-02

**Authors:** 


### The Doctor's Armchair.

"DIED OF ACUTE PNEUMONIA."

"The many friends of——will regret to learn of his sudden death at the early age of forty-six of acute pneumonia." This, and similar announcements, have been so exceedingly frequent in the daily papers during the last few months that numbers of people are beginning to be seriously alarmed. What is pneumonia, and why does it seem to be killing so many men, in the very prime of manhood, during the present spring? By way of partial answer to this question a significant paragraph upon "Atmospheric Influences" may be quoted from *Quain's Dictionary of Medicine.* "Conditions of weather and climate," says Dr. Thomas Henry Green in *Quain,* "are probably the most important of all known agencies in the causation of pneumonia. The influence of cold and damp in increasing the liability to acute inflammatory diseases of the chest is well known. This influence is marked in pneumonia, although to a much less extent than in bronchitis. Pneumonia is more common in temperate climates than in those regions which are characterised by great heat or extreme cold. Climates and seasons which are liable to sudden changes of temperature, and winds from the north and north-east, appear to be especially favourable to this disease."

Environment, then, is chargeable with some portion of the blame which attaches to the destruction of certain men's lives in middle age. But it is not to environment alone that we must look; the men themselves must be considered, with the amount of their health knowledge, and the use they make of it. Climate as a part of environment, may, however, properly be considered first. No soldier can be said to be reasonably sure of victory until he understands the tactics of his enemy, and is prepared either to anticipate or to defeat them. What great lawyer was it of whom it was said that "he never made the mistake of treating his adversary with contempt"? Englishmen constantly make the mistake of treating their adversary, the English climate, with contempt, and they constantly pay for their error with their lives. The keen winds of spring, which Hood so well knew as the "bleak winds of March," are exceedingly formidable. They shift from north to east, from east to west, and from west back to north again, and to every other conceivable point of the compass with an uncertainty which is bewildering and a celerity which is astonishing. They raise the temperature from thirty-five to sixty in an hour, and depress it from sixty to thirty-five in a quarter of that time. They make a heavy dragoon charge at a gallop full in your face at one moment, and before you can defend yourself against that they wheel round and attack your liver on your right side, or your lung on the left, or your kidneys in the rear. If they do not succeed in setting up a liver fever or a smart pleurisy, they will inflict upon your legs an agonising sciatica, or upon your head a stinging neuralgia. If they be accompanied by heavy rains, or even by frequent flying showers, they make the atmosphere moist as well as cold, and thus chill the inside of the lungs as well as the surface of the body. Under such circumstances they often succeed in their malignant design of leaving a fatal pneumonia behind.

Will anyone deny that the March and April winds of this country are such as we have described? And will anyone affirm that such enemies can be treated with contempt? The Scotch minister who was giving a pre-matrimonial exhortation to a young couple by way of dissuader, said, "My friends, marriage is a blessing to few, a curse to many, and a trouble to all; do ye venture?" The young couple did venture. Now, it is possible that the keen winds of March and April may be a blessing to a very few; but it is certain that they are a curse to many, and more or less of a trouble to all. Still, there is no way of escaping them unless we desert our country; and we shall not desert Britain at the bidding of the spring winds. We must therefore "venture."

This brings us to the second part of our contention. Seeing that we must venture, we ought to venture intelligently, and with adequate preparation. North country folk have an old but somewhat rough and ready method of fighting the piercing blasts of spring. It is laid down in the rule which is thus expressed: "Till May be oot, cast na a cloot." This, although by no means so good a rule as one would desire, is yet, very much better than no rule at all. It is like advising soldiers always to fight within their entrenchments. Soldiers, so advised, it is evident, are not likely to make any very extensive conquests of new territory. But will you give us a better rule? the north countryman may ask. To which it is replied, that elementary rules are for elementary people, and elementary conditions of life. Now, we in Great Britain are not elementary people, and we certainly do not live under elementary conditions of life. It is imperative upon us to live rather by the daily exercise of instructed reason than by unthinking obedience to mechanical rules.

Let nobody think that these phrases are mere doctors' platitudes. Many persons do give doctors credit for profuse platitudinarianism. Such persons ought not to be surprised if the doctor sometimes feels that they are right served when they suffer for despising his instructions. Even so great a poet as Dante could not hide a certain grim satisfaction on seeing some of his old opponents working out their immortal destiny in what, to say the least, was a considerable degree of discomfort. The doctor is but mortal; and he puts this little preface to the application of his discourse, because he wants his readers to think of what is said, and to carry it out in practice if it convinces their reason.

Two sorts of preparation are required for meeting successfully the cunning, furious, and almost always dangerous onslaughts of the spring winds. The first is what may be called internal preparation; the second is external preparation. The chief internal preparation is that of eating regular, sufficient, and suitable meals. Mere regularity in eating is one of the strongest of all defences against sudden attacks of illness. Of course, if we are to eat regularly, we must have appetites; and whoever finds his appetite failing in the spring should seek medical aid immediately. For his appetite is the strongest of all his inner defences; and when that is gone the enemy can rush in at pleasure. Regularity of meals, which includes regularity of work, is the chief of all internal preparations. External preparation against the spring winds consists in a sufficient variety of clothing. On winter days, even in March and April, winter overcoats, boots, stockings, underflannels, and even winter gloves must be worn. For warmer days, when the wind is west or south, and the sun shines, clothing a little lighter, but still of wool, must be worn throughout. But above all, a spring overcoat for men, buttoned up as closely as the winter overcoat, must still be used. It may be of much lighter material, and it should be made a little looser and easier than the winter overcoat; but on no account should it be dispensed with until full summer has arrived. The overcoat is the great outwork of external preparation that prevents the chill or damp wind from ever approaching dangerously near the body. Perhaps, the writer ought to apologise for giving these directions in the imperative mood, and for terminating them a little abruptly. But, it may be admitted as a sufficient excuse that the reasons of the rules will be found in experience, and that the neglect of them is dangerous. Every thinking man can easily prove all these things by personal experimentation.

